# Telangiectatic adenoma – computed tomography and magnetic resonance findings: a case report and review of the literature

**DOI:** 10.1186/1757-1626-2-24

**Published:** 2009-01-07

**Authors:** Tatiana Chinem Takayassu, Edson Marchiori, Antonio Eiras, Rafael Ferracini Cabral, Fernanda Caseira Cabral, Raquel Ribeiro Batista, Gláucia Zanetti, Paula Cristina Pereira Dias

**Affiliations:** 1 Department of Radiology of the Federal University of Rio de Janeiro. Rua Professor Rodolpho Paulo Rocco, 255. Cidade Universitária. CEP 21941-913, Brazil; 2 Department of Radiology of the Fluminense Federal University. Rua Marquês do Paraná, 530. Centro. CEP 24000-000 Niterói. Rio de Janeiro. Brazil; 3 Department of Radiology, Hospital Barra D'Or, LABS-D'Or Hospital Network, Avenida Ayrton Senna, 2541. CEP 22775-002, Barra da Tijuca. Rio de Janeiro, Brazil

## Abstract

Telangiectatic adenoma is a new classification of a hepatic lesion. It was previously named telangiectatic focal nodular hyperplasia but it is in fact true adenoma with telangiectatic features. We report here a case of telangiectatic adenoma in a 72-year-old woman. The image features are lack of a central scar, a heterogeneous lesion, hyperintensity in T1-weighted MR images, strong hyperintensity in T2-weighted MR images, and persistent contrast enhancement in delayed-phase contrast-enhanced CT or T1-weighted MR images. It is a monoclonal lesion with potential of malignancy. The treatment of telangiectatic adenoma is surgery, the same way as hepatic adenoma. Focal nodular hyperplasia may be managed by clinical follow-up alone.

## Background

Telangiectatic adenoma (TA), previously known as telangiectatic focal nodular hyperplasia, was recently redefined as a variant of hepatocellular adenoma (HA), based on molecular and genetic evidence. This redefinition has potential clinical relevance because telangiectatic adenomas must be as aggressively managed as hepatocellular adenomas. Here we describe a case of a female patient presenting with a liver mass which turned out to be TA, and discuss this new pathological entity and its differential diagnosis, with emphasis on interpretation of imaging results.

## Case presentation

A 72-year-old asymptomatic woman presented to the clinic for a checkup. She had a history of cholecystectomy, hypothyroidism, and hypertension. She had been treated with thyroid hormone (Puran^® ^T4, 75 μg), an anti-hypertensive agent (the angiotensin-converting enzyme inhibitor Enalapril^®^, 20 mg), an antiarrhythmic drug (Diltiazem^®^, 90 mg), and acetylsalicylic acid (200 mg). She reported use of oral contraceptives for over 30 years. She had no history of smoking, drinking, drug abuse or family history of liver disease.

On physical examination, the liver was palpable to 6.5 cm from the right costal, and 10 cm from the appendix xiphoid; it was painless, and had a rugged and hard consistency. Laboratory studies revealed the following: red blood cells, 3,690,000/mm^3^; hemoglobin, 11.7 g/dL; hematocrit, 35%; platelets, 278,000/mm^3^; cholesterol, 186 mg/dL; triglycerides, 70 mg/dL; glucose, 85 mg/dL; TSH, 2.8 U/mL; serum creatinine, 0.7 mg/dL; ALT, 47 U/L; AST, 29 U/L; GGT, 23 U/L; alkaline phosphatase, 138 U/L; TB, 0.8 mg/dL; DB, 0.4 mg/dL; PT, 12 s; INR, 1.0; PTT, 29 s; folic acid, 11 mg/mL; alpha-fetoprotein, 2.08 ng/mL; iron, 106 μg/dL; vitamin B12, 210 pg/mL; ferritin, 170 mg/L; LDH, 384 IU/L; and CEA 2.4 ng/mL.

Abdominal ultrasound revealed an enlarged and heterogeneous liver that contained an expansive and heterogeneous lesion measuring 14 × 9.4 cm with poorly defined limits, including two echogenic nodular areas, measuring 6.3 and 3.2 cm, respectively. The gallbladder was not visualized.

**Figure 1:**
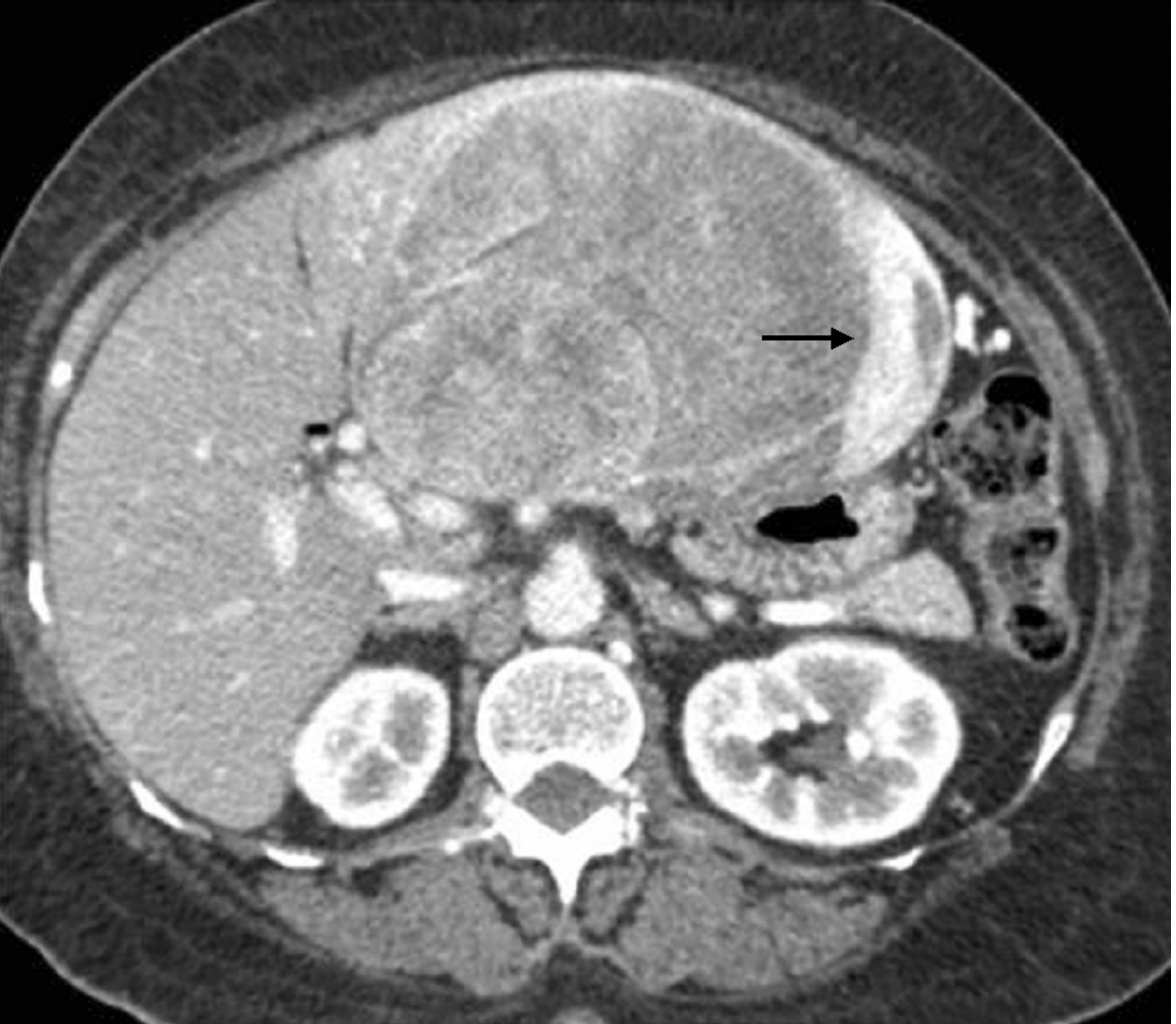
**Portal-stage contrast-enhanced helical CT of the upper abdomen**. Impregnation by the contrast agent shows a large, solid, exofitic lesion compromising the left liver lobe. The enhancement is intense and heterogeneous, the dominant portion being in the periphery of the lesion (arrow).

Computed tomography (CT) of the upper abdomen (Figure 1) showed a heterogeneous lesion – measuring 15 × 14 × 10 cm – with a partially well-defined margin and hypodense permeating areas, some with attenuation values compatible with soft parts, and others with liquefaction located in the left liver lobe. After endovenous contrast injection, the lesion showed intense and non-homogeneous impregnation in the arterial and portal phases, with hypodense permeated areas located in the left lobe, notably parts II and III; these areas determined recoil and reduced the size of vascular structures adjacent to the liver. The injury also caused bulging of the border of adjacent liver tissue, causing a compressive effect on other abdominal structures, especially the stomach and pancreas.

**Figure 2:**
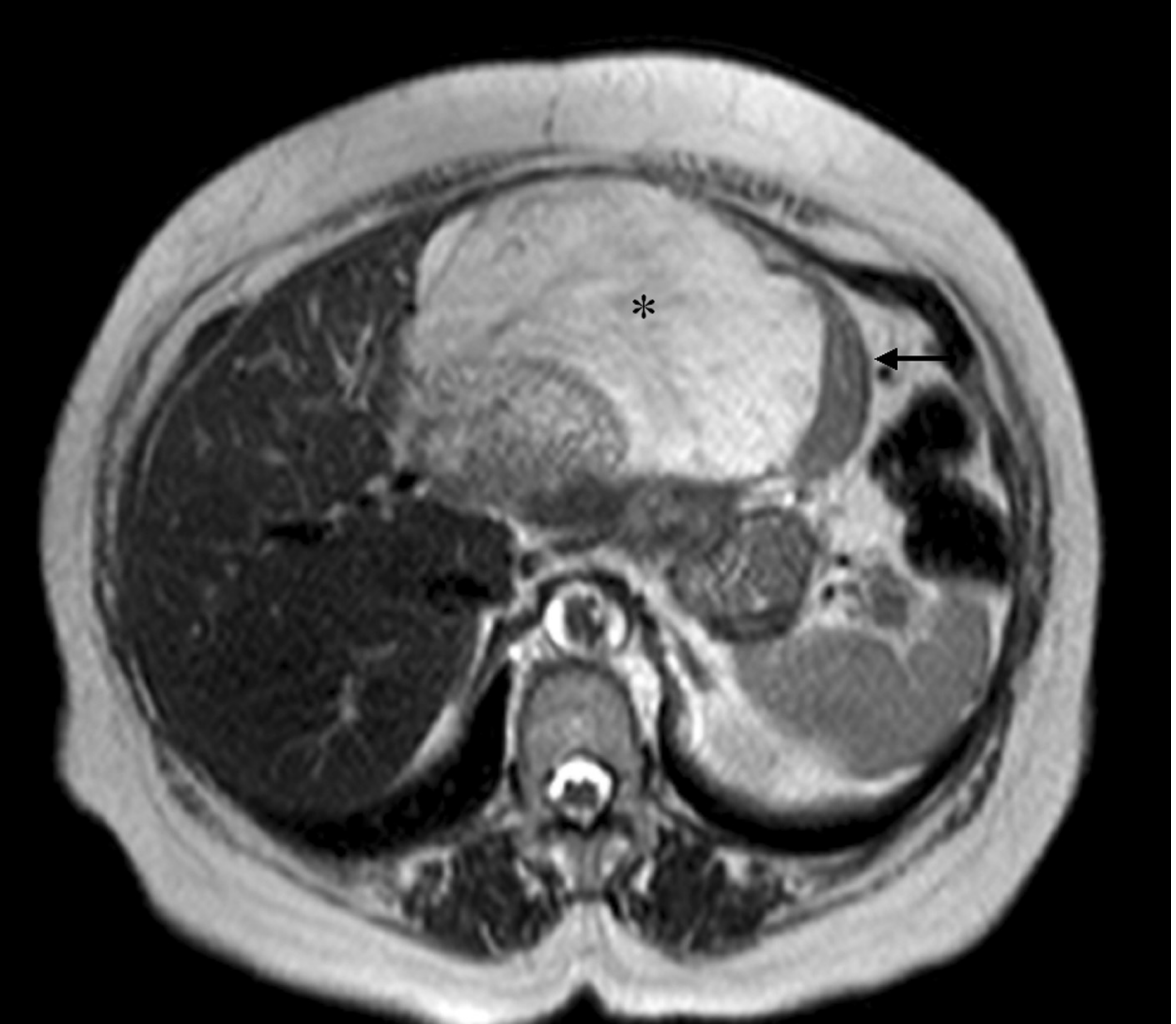
**MR imaging sequence with T2-weighted transversal**. Imaging shows a hypointense signal originating in the peripheral region (arrow) and a dominant component associated with a hyperintense signal (*).

**Figure 3:**
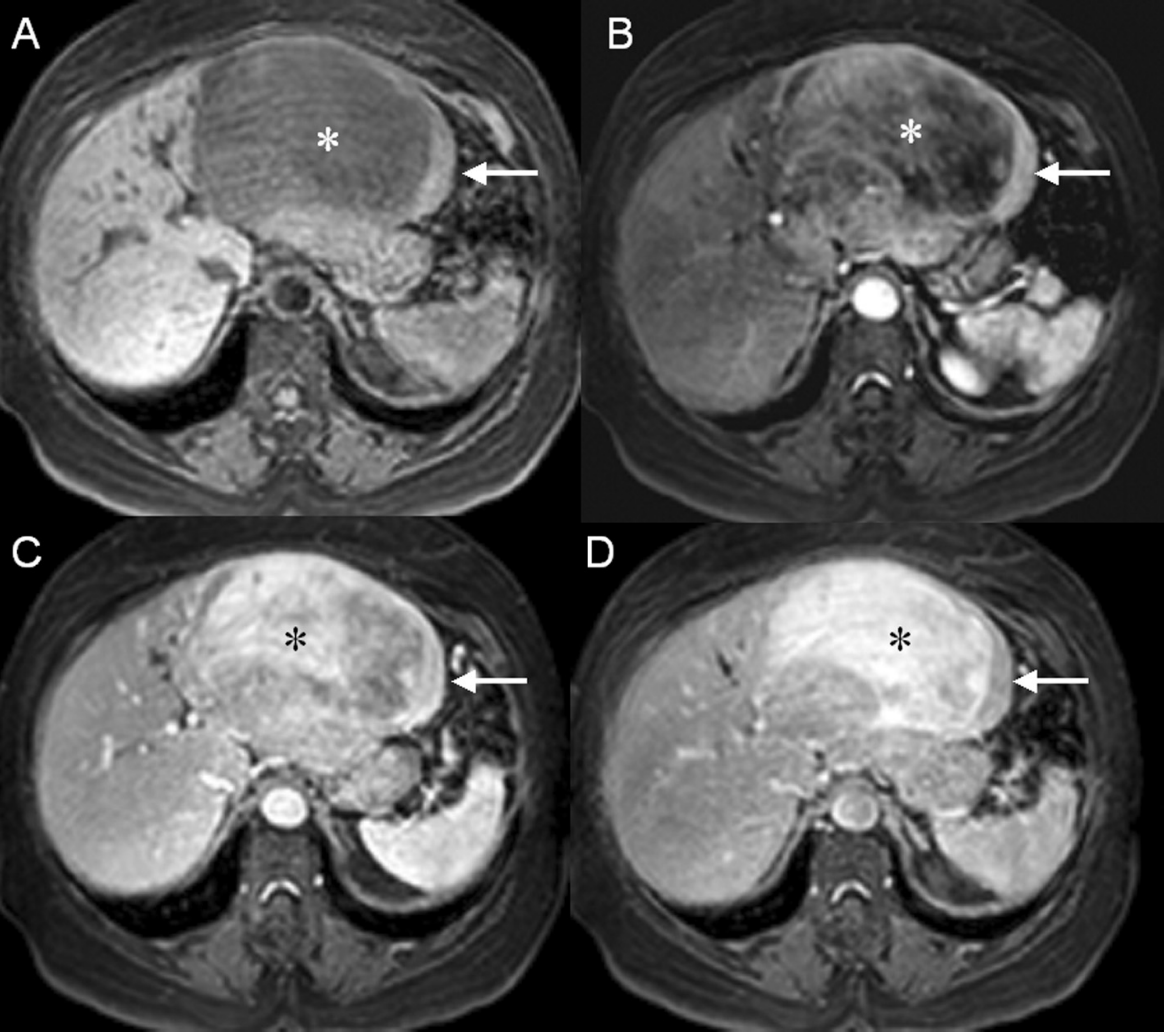
**(A) T1-weighted fat-suppressed MR imaging without contrast; and MR imaging after contrast in (B) arterial phase, (C) portal phase, and (D) late phase**. In T1-weighted images without contrast, the signal in the periphery of the mass (arrow) is similar to the muscle signal; the center of the lesion clearly shows a hypointense signal (*). Although impregnation of both components began in the arterial phase, the peripheral region (arrow) showed a stronger signal during the arterial and portal phases, followed by signal decay in the late phase due to relatively rapid washout, indicative of hypervascularization. The enhancement of the central component is far more intense in the late phase (*), probably due to a large interstitial space and slow flow through the vascular components.

Magnetic resonance (MR) imaging of the upper abdomen showed different components within the lesion. T2-weighted images in the peripheral region showed a discrete heterogeneous hyperintense signal, while the central portion was hypointense. In T1-weighted images, the signal in the periphery of the mass was similar to the muscle signal and the center of the lesion showed a signal that was clearly hypointense. Although impregnation of both components began in the arterial phase, the peripheral region showed a stronger signal during the arterial and portal phases, followed by signal decay in the late phase due to relatively rapid washout, indicative of hypervascularization. The center of the mass was far more intense in the late phase, probably due to slow flow through the vascular components (Figures 2 and 3).

Surgery and pathological analysis revealed a hepatic segment measuring 17.5 × 14 × 8 cm and weighing 1200 g that contained a 15 × 9 cm brown tumor with ill-defined limits. The tumor had a gelatinous component and showed hemorrhaged, necrotic, and cystic areas. The final diagnosis was TA. The patient progressed well and she remains asymptomatic after one year of follow up.

## Discussion

Focal nodular hyperplasia (FNH) and liver cell adenoma (LCA) are the most frequent benign epithelial lesions of the liver observed in young female patients using oral contraceptives. Although they are both benign, the management of these two types of lesions is radically different, consisting of clinical follow-up for FNH and surgical resection for liver cell adenoma [1,2].

FNH is the second most common benign liver tumor, occurring predominantly in young and middle-aged women [3,4]. The tumor-like lesions probably result from a hyperplastic liver response to a focal blood flow increase related to pre-existing arterial malformation [4,5]. Their polyclonal nature suggests that they are part of a regenerative process; consequently, they are unlikely to present a risk of malignancy [1].

FNH is usually asymptomatic and is often discovered fortuitously. A non-operative approach has been adopted by most hepatobiliary centers because there are no proven cases of malignant degeneration. Pain may sometimes be present, particularly in the case of a large tumor, but hemorrhage is very rarely reported [6]. Unlike adenomas, 70% of cases of FNH may be diagnosed by imaging techniques alone [2]. The recommended follow-up consists of serial ultrasonography. The main goal of imaging in FNH patients is to firmly establish the diagnosis in order to avoid surgical resection and to confirm that a conservative approach to therapy is appropriate [7,8].

LCA is a rare hepatic tumor that is characterized pathologically by the benign proliferation of hepatocytes [4]. These tumors are strongly associated with oral contraceptive use [9]. Distinction between LCA and FNH is not possible based only on clinical and laboratory data.

LCA usually comprises a single nodule; more rarely, multiple nodules may be present [10,11]. In contrast to regenerative lesions such as typical FNHs, LCAs are monoclonal tumors, suggesting that they display neoplastic rather than regenerative behavior; consequently, they may present a risk of malignancy [1]. LCAs are usually symptomatic, presenting with abdominal pain or abnormal liver function tests. Surgical resection is advocated based on the high incidence of bleeding complications and reports of neoplastic degeneration [8]. Because of these risks of spontaneous rupture and malignant transformation, LCAs must be identified and treated promptly.

Based on previous studies, it is now clear that the lesions previously named telangiectatic focal nodular hyperplasias are in fact true adenomas with telangiectatic features; hence they are now referred to as telangiectatic adenomas (TAs). This new classification is based on both morphological and molecular features, and takes into consideration the results of clonal analysis and gene expression studies. It should be emphasized that the new classification has clinical relevance; aggressive management of these cases should be considered. However, it remains to be established whether the clinical behavior of TAs, including the associated risk of malignant progression, resembles that of LCA more than FNH.

TA is defined as a benign well-differentiated proliferation of hepatocytes in which vascular changes, including telangiectatic features, are prominent [9]. Clonal analysis of TA showed that most examples displayed an X-chromosome inactivation pattern consistent with monoclonality. This monoclonal nature of TAs suggests that they display neoplastic rather than regenerative behavior; therefore, unlike regenerative lesions such as typical FNHs, they may present a risk of malignancy [1]. Diagnosis of a TA requires the observation of at least 4 of the following features: lack of a central scar, a heterogeneous lesion, hyperintensity in T1-weighted MR images, strong hyperintensity in T2-weighted MR images, and persistent contrast enhancement in delayed-phase contrast-enhanced CT or T1-weighted MR images [7].

Benign hepatic tumors represent a broad spectrum of regenerative and true neoplastic processes. Many of these tumors present with characteristic features in imaging studies. The new classification of TAs has clinical relevance because they must be aggressively managed in the same way as HAs, whereas FNH may be managed by clinical follow-up alone.

## Abbreviations

FNH: focal nodular hyperplasias; LCA: liver cell adenoma; TA: telangiectatic adenomas

## Consent

Written informed consent was obtained from the patient for publication of this case report and any accompanying images. A copy of the written consent is available for review by the Editor-in-Chief of this journal.

## Competing interests

The authors declare that they have no competing interests.

## Authors' contributions

TCT conceived the study. RFC, FCC and RRB performed the literature review. TCT, AE, GZ and EM edit and coordinated the manuscript. All authors read and approved the final manuscript.
